# Novel type of medial meniscus ramp lesion: a case report and surgical technique

**DOI:** 10.1093/jscr/rjab538

**Published:** 2021-12-07

**Authors:** Abdulaziz Z Alomar

**Affiliations:** Division of Arthroscopy & Sports Medicine, Department of Orthopedic Surgery, King Saud University, Riyadh, Kingdom of Saudi Arabia

## Abstract

Meniscal ramp lesions have been reported in 9–24% of patients who underwent anterior cruciate ligament reconstruction (ACLR). We report a rare type of double medial meniscus ramp lesion in in a 26-year-old male soccer player who presented with persistent knee instability and an inability to return to sports after a successful ACLR due to unaddressed and untreated ramp lesions. To the best of our knowledge, this is the first reported case of a double ramp lesion, with tears occurring in two separate locations: one tear at the meniscosynovial junction and associated with meniscotibial ligament disruption; and a second, more posteriorly located at the meniscocapsular junction and associated with meniscocapsular attachment disruption. It was found to be very unstable upon arthroscopic assessment and was clinically associated with persistent knee instability even after ACLR, thus necessitating surgical repair to restore knee kinematics.

## INTRODUCTION

In 1988, a specific type of meniscal injury in which an ACL injury involving the peripheral attachment of the posterior horn of the medial meniscus (PHMM) was described [1]. This phenomenon was later termed a ‘ramp lesion’, an injury characterized by the presence of a tear at the peripheral meniscocapsular attachments of the PHMM [2]. Ramp lesions have previously been reported in 9–24% of patients who underwent ACL reconstruction [3–6]. However, current literature has defined ramp lesions as tears in the meniscocapsular ligament, meniscotibial ligament (MTL) or both [7].

Ramp lesions are typically classified using a standard approach based on their arthroscopic morphology [6, 8]. Herein, we report a rare type of novel double medial meniscus ramp lesion in which one tear occurred at the meniscosynovial junction and was associated with meniscotibial ligament disruption and a second, more posteriorly located tear was found at the meniscocapsular junction and was associated with meniscocapsular attachment disruption. To the best of our knowledge, this is the first reported case of a double ramp lesion occurring in two separate locations, one of which was outside the meniscal substance of the PHMM.

## CASE PRESENTATION

A 26-year-old male soccer player presented to the clinic 8 months after having undergone an isolated ACLR. The patient complained of persistent knee instability during sporting activities and an inability to return to sports.

Upon examination, the affected knee showed grade 1 laxity during the anterior drawer test, albeit with a solid end-point. Otherwise unremarkable knee examination.

**Figure 1:**
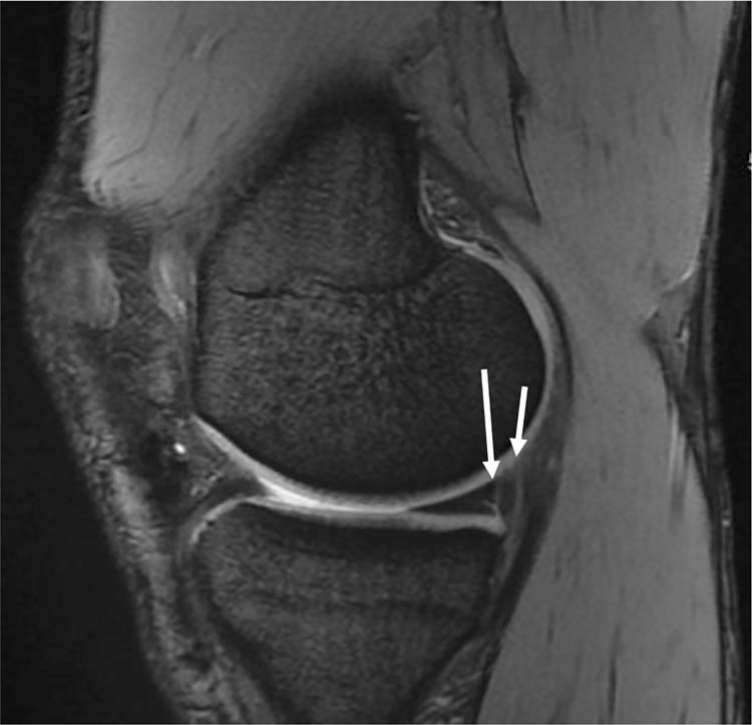
Sagittal magnetic resonance imaging of the knee. Gradient echo sequence image of the medial compartment of the knee demonstrating a suspicious double ramp lesion. The long arrow indicates the first ramp lesion, and the short arrow indicates the second ramp lesion

Radiography revealed normal findings. MRI revealed an intact ACL graft with proper tunnel positions. A ramp lesion was suspected based on the findings of MRI (Fig. 1).

**Figures 2–4:**
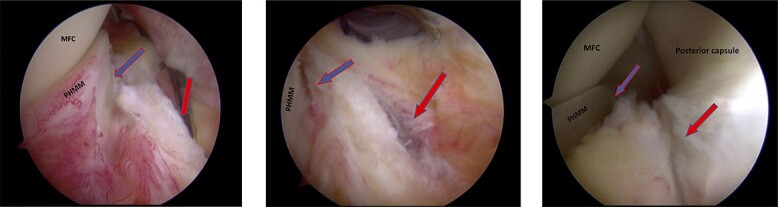
Trans-notch view of the posteromedial compartment of the left knee showing the blue arrow indicates the first lesion at the meniscosynovial junction of the red-red zone, and the red arrow indicates the second tear at the meniscocapsular attachment.

### Surgical technique and postoperative rehabilitation

**Figures 5–6:**
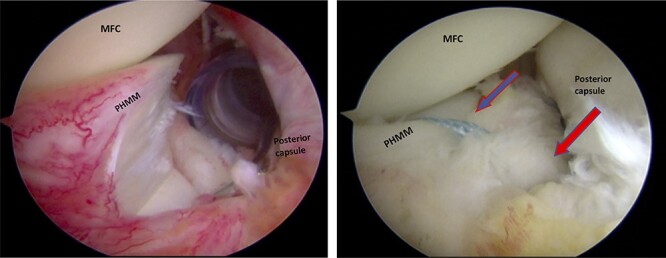
Trans-notch view of the posteromedial compartment of the left knee showing the surgical repair of the first tear. The blue arrow indicates the first lesion at the meniscosynovial junction in the red-red zone area. The red arrow shows the second tear at the meniscocapsular attachment. PHMM, posterior horn of the medial meniscus; MFC, medial femoral condyle

Standard diagnostic arthroscopy confirmed the presence of an intact ACL graft. Trans-notch arthroscopic visualization revealed a double meniscus ramp lesion (Figs 2–5). It was noted that the tear located at the meniscosynovial junction in the red-red zone of the PHMM was associated with MTL disruption, while the second tear was more posteriorly situated at the meniscocapsular junction and was associated with meniscocapsular attachment disruption. Probing of the meniscus resulted in its significant displacement.

**Figure 7:**
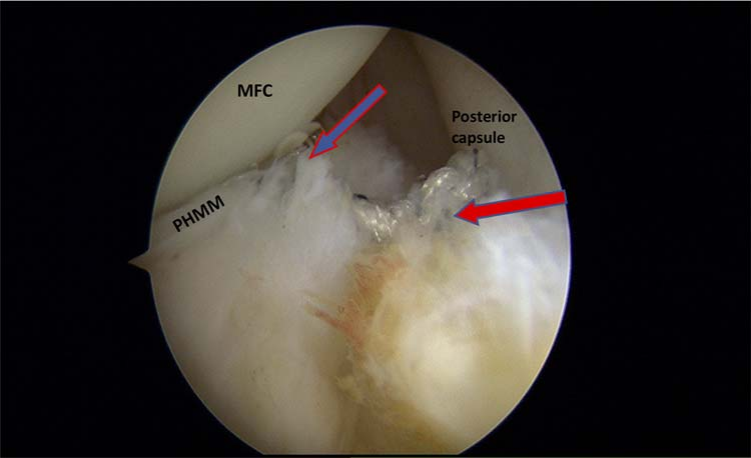
Trans-notch view of the postromedial compartment of the left knee showing the repair of both ramp lesion tears. The blue arrow indicates the repair of the first lesion at the meniscosynovial junction of the red-red zone area. The red arrow indicates the repaired second tear at the meniscocapsular attachment. PHMM, posterior horn of medial meniscus; MFC, medial femoral condyle

A posteromedial portal was utilized to repair both ramp tears using all-inside sutures, and curved suture hooks were inserted through the posteromedial portal. The first tear was repaired, and a non-absorbable suture was passed between the peripheral edge of the PHMM and attachment of the meniscotibial ligament (Fig. 6). The second tear was then repaired by passing the suture through the posterior capsular tissue, creating a bridge between the two tears to restore the meniscocapsular attachment (Fig. 7).

**Figure 8:**
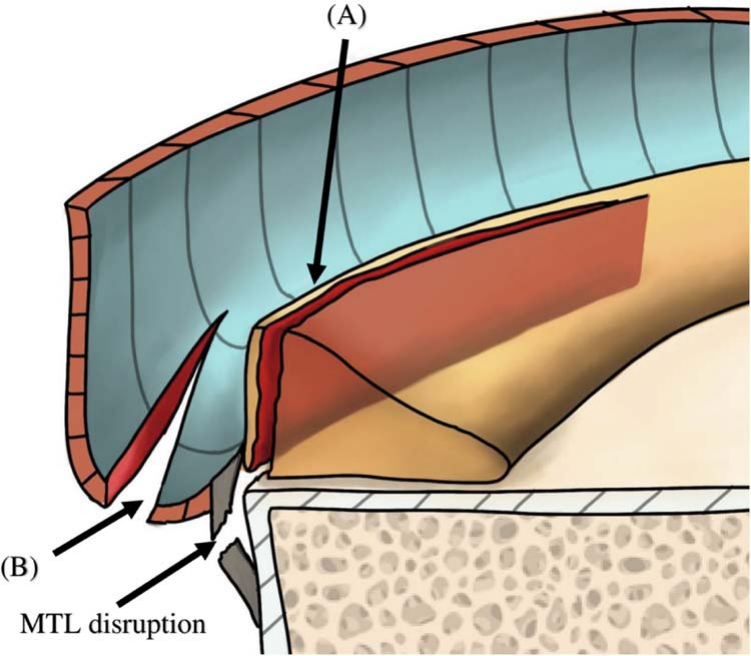
Illustration of the sagittal view of the posteromedial compartment showing the morphology of the tears. Panel (**A**) indicates the first tear, which was located at the PHMM of the meniscosynovial junction and was associated with disruption of the MTL. Panel (**B**) indicates the second tear, which was located at the meniscocapsular junction and was associated with disruption of the meniscocapsular attachment to the PHMM. PHMM, posterior horn of medial meniscus; MTL, meniscotibial ligament.

The rehabilitation on first 2 weeks focused on edema control, knee range of motion and quadriceps-activation exercises. Protected weight-bearing was advised for the first four postoperative weeks. The patient was able to successfully resume sporting activities after six postoperative months. The last follow-up was 3 years after surgery, and the patient had no symptoms and could play soccer normally.

## DISCUSSION

When unstable ramp lesions are missed, the ACL graft may be subjected to increased stress, ultimately leading to ACL failure [9–11]. Other possible consequences include increased tibiofemoral contact pressure and a reduced contact area [9, 12]. Ramp lesion should be suspected in patients with high-grade Lachman and pivot-shift test results in the presence of an ACL tear and in those with persistent instability following ACLR [9].

In the present, case ramp lesion was found to be highly unstable, as both the meniscocapsular and meniscotibial attachments had been damaged. These clinical findings were consistent with those of a previous report by DePhillipo *et al*. on cadavers, which assessed the biomechanical effects of MTL and meniscocapsular attachment tears. Such tears can increase knee anterior tibial translation and pivot shift in ACL-deficient knees. Furthermore, they found that the pivot shift was restored only when ACL-R performed concomitantly with meniscocapsular and meniscotibial repair [9].

DePhillipo *et al*. [7] found that the meniscocapsular and meniscotibial ligaments merged together at a common attachment site connecting to the PHMM. For this reason, a tear at the common attachment, otherwise referred to as the meniscosynovial junction, can result in disruption of both ligaments. In the present case, repairing the first tear resulted in reattachment of the common attachment to the PHMM, which, in turn, secured the attachment of the MTL. Nonetheless, this approach cannot restore the meniscocapsular attachment in the presence of a second, posteriorly located tear.

Thaunat *et al*. [8] classified ramp lesions into five types according to their arthroscopic morphology: The first four types are single tear lesions. The fifth type of ramp lesion includes double tears that involve the PHMM at the meniscosynovial junction as well as a second, anteriorly located tear at the PHMM. Based on our findings, we propose that the aforementioned classification be modified to include a new class of tear. The double ramp lesion described in the current report differs from that described in the Thaunat classification, in which the first tear is located in the meniscosynovial junction at the red-red zone of PHMM and is associated with MTL disruption, whereas the second tear is posteriorly located at the meniscocapsular junction and is associated with meniscocapsular attachment disruption (Fig. 8). Therefore, our modification to the Thaunat type 5 (double ramp lesion) should subdivided into subtype A (the previously described by Thaunat *et al*.) and subtype B (our newly described lesion).

In the present case, MRI was ambiguous in diagnosing the double ramp lesion, possibly due to the fact that the MRI was performed with the knee in full extension. It has been reported that identification of ramp lesions may be hampered by performing MRI with the knee in full or near full extension, as this position decreases the meniscocapsular separation [13, 14].

The decision as to whether ramp lesions require surgical or non-surgical repair remains controversial. Those who advocate for the use of surgical repair of all meniscal ramp lesions at the time of ACL reconstruction generally do so based on the increased risks of persistent knee instability and ACL graft failure posed by leaving ramp lesions untreated [2, 15].
